# Upsurge of Enterovirus D68, the Netherlands, 2016

**DOI:** 10.3201/eid2301.161313

**Published:** 2017-01

**Authors:** Marjolein Knoester, Elisabeth H. Schölvinck, Randy Poelman, Sylvia Smit, Clementien L. Vermont, Hubert G.M. Niesters, Coretta C. Van Leer-Buter

**Affiliations:** University Medical Center Groningen, Groningen, the Netherlands

**Keywords:** enterovirus, infectious myelitis, flaccid quadriplegia, respiratory insufficiency, infectious disease outbreaks, viruses, the Netherlands, EV-D68

## Abstract

In June and July 2016, we identified 8 adults and 17 children with respiratory enterovirus D68 infections. Thirteen children required intensive care unit admission because of respiratory insufficiency, and 1 had concomitant acute flaccid myelitis. Phylogenetic analysis showed that all of 20 sequences obtained belong to the recently described clade B3.

The largest enterovirus D68 (EV-D68) outbreak occurred in the United States during the summer and fall of 2014. Approximately 1,100 respiratory EV-D68 infections were recorded, mostly in children, many of whom required intensive care unit (ICU) admission. Most diseases were of respiratory nature, but concurrent with the upsurge of EV-D68, the Centers for Disease Control and Prevention (Atlanta, GA, USA) recorded 120 cases of acute flaccid myelitis (AFM) (*1*). A Europewide study identified circulation of EV-D68 during the same period. Although numbers were lower, ICU admissions and 3 cases of AFM were reported (*2*). Before 2014, only a few small outbreaks of EV-D68 had been described worldwide, all with respiratory infections.

Our clinical virology laboratory (University Medical Center Groningen, Groningen, the Netherlands) reported an increase of EV-D68 infections in 2010 and again in 2014, in parallel with the US outbreak (*3*,*4*). Very limited activity of EV-D68 was observed in 2015. Since June 2016, we have again identified a substantial increase in respiratory EV-D68 infections in our hospital, just 2 years after the previous upsurge. To raise awareness of this upsurge and the severity of EV-D68 infections, we report on 25 cases. In addition, we show the phylogenetic relationship between the 2016 EV-D68 strains and those that circulated in 2014.

## The Study

The University Medical Center Groningen is a tertiary referral center in the northern part of the Netherlands. We perform routine diagnostic real-time PCR for all patients evaluated for respiratory disease by laboratory developed tests for 17 targets, including adenovirus, bocavirus, coronavirus, enterovirus, metapneumovirus, influenza, parainfluenzavirus, rhinovirus, and respiratory syncytial virus (*5*). From all enterovirus detections with a cycle threshold (C_t_) value <31, we sequence part of the viral protein 1 gene on a weekly basis (*6*). To obtain rapid typing results in the current outbreak, a specific EV-D68 real-time PCR was also used (*3*), when enterovirus PCR was found positive.

**Figure 1:**
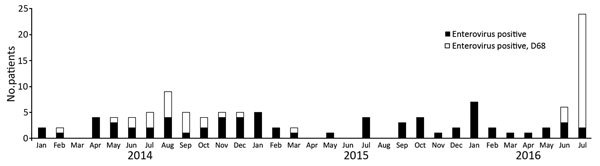
Respiratory enterovirus infections, University Medical Center Graningen, the Netherlands, 2014, 2015, and 2016. The total numbers of respiratory specimens tested for enterovirus in these years during April–July were 784, 828, and 871, respectively. Seventeen (2.2%) of 784 were positive for enterovirus in 2014, 5 (0.6%) of /828 in 2015, and 33 (3.8%) of /871 in 2016. Six (35%) of 17 were typed as enterovirus D68 in 2014, versus 0 of 5 in 2015, and 25 (76%) of 33 in 2016.

We identified 3 EV-D68 infections in June and 22 additional cases in July. We found a stable seasonal variance for enterovirus and a sudden rise of enterovirus infections in July, mainly caused by EV-D68 (Figure 1).

The EV-D68–positive specimens were nasopharyngeal swabs (n = 8), sputum (n = 2), and nasopharyngeal brushes (n = 15). Two viral respiratory co-infections were detected: rhinovirus A15 in patient 12 (enterovirus C_t_ 17, rhinovirus C_t_ 21) and parainfluenzavirus type 4 in patient 23 (enterovirus C_t_ 14, parainfluenza virus 4 C_t_ 31). For 20 patients, we obtained sequence results identifying EV-D68; the remaining 5 could not be typed by sequencing but were positive in our specific EV-D68 PCR.

Of the 25 patients, 8 were adults and 17 were children (Table). In the adults, 6 of whom were transplant recipients, symptoms were mild and influenza-like. In children, however, we observed life-threatening respiratory distress, for which ICU admission was necessary for 13 (Table). Twenty-one patients had an underlying or concurrent condition, in the children mostly pulmonary.

**Table:** Characteristics of patients with EV-D68 infection, University Medical Center Groningen, Groningen, the Netherlands, June 11– August 1, 2016*

Patient	Date†	Age/sex	Concurrent condition(s)	Clinical characteristics	ICU admission, days in ICU
1	Jun 11	1 y /M	None	Bronchial obstruction, respiratory failure	Yes/4
2	Jun 20	3 y/M	None	Status asthmaticus	Yes/3
3	Jun 30	61 y/F	Lung and liver transplantation	Cough, runny nose	No
4	Jul 4	5 y/M	Asthma	Status asthmaticus	Yes/2
5	Jul 5	3 y/M	Asthma	Status asthmaticus	Yes/3
6	Jul 7	67 y/F	Autologous stem cell transplantation	Influenza-like syndrome, dyspnea	No
7	Jul 7	2 y/M	Pulmonary hypertension, underdeveloped lung vessels	Respiratory failure	Yes/4
8	Jul 11	70 y/F	Allogenic stem cell transplantation	Influenza-like syndrome, dyspnea	No
9	Jul 14	66 y/F	Allogenic stem cell transplantation	Cough, fever	No
10	Jul 16	3 y/M	Asthma	Status asthmaticus	Yes/4
11	Jul 16	3 mo/M	Bronchopulmonary dysplasia of prematurity	Respiratory failure	Yes‡
12	Jul 16	1 y/F	Bronchial hyperreactivity, failure to thrive	Bronchial obstruction, respiratory failure	Yes/8
13	Jul 22	5 y/F	Asthma	Status asthmaticus	Yes/5
14	Jul 22	1 y/M	Tracheal stoma for bilateral vocal cord paralysis	Cough, wheezing	No
15	Jul 23	3 y/M	None	Status asthmaticus	Yes/5
16	Jul 24	3 y/M	Atypical seizures 3 y prior	Acute flaccid myelitis	Yes/ongoing§
17	Jul 25	19 y/F	None	Influenza-like syndrome	No
18	Jul 25	3 y/M	Bronchial hyperreactivity	Asthma exacerbation	No
19	Jul 25	5 mo/M	22q11 deletion, tetralogy of Fallot, tracheal stoma	Acute mechanical airway obstruction, resuscitation	No¶
20	Jul 28	50 y/M	Lung transplantation	Cough, runny nose	No
21	Jul 28	3 y/M	Spinal muscular atrophy	Respiratory failure	Yes/5
22	Jul 29	9 mo/M	Corrected esophagal atresia with related tracheomalacy for which noninvasive ventilation at home	Respiratory distress with bronchorrhea	No
23	Jul 30	3 y/F	Bronchial hyperreactivity	Status asthmaticus	Yes/2
24	Jul 30	51 y/F	Autologous stem cell transplantation	Influenza-like syndrome	No
25	Jul 31	23 y/M	Childhood asthma	Cough, dyspnea, nausea, vomiting	No

*EV, enterovirus; ICU, intensive care unit. †Date of first EV-D68–positive specimen. ‡Patient 11 contracted EV-D68 in the neonatal ICU. §Patient still in ICU as of December 6, 2016.  ¶Patient 19 acquired EV-D68 while admitted to the special care ward since birth.

One child (patient 16) had the clinical characteristics of AFM. This nearly 4-year-old boy had a history of headaches for 1 week and an influenza-like illness for 3 days. He had rapidly progressing asymmetric weakness of arms and legs, bulbar paralysis, asymmetric facial paralysis, and respiratory distress for which he needed ventilatory support. Cerebrospinal fluid (CSF) analysis showed no abnormalities. Axial fluid-attenuated inversion recovery magnetic resonance imaging (MRI) showed hyperintense nonenhancing gray matter lesions in brainstem and spinal cord. Electromyography findings supported injury on the level of the motor axon or the anterior horn of the spinal cord. No varicella zoster virus, herpes simplex virus, enterovirus, or parechovirus were detected in the CSF. In the nasopharyngeal swab, only EV-D68 was detected.

All patients in this report were treated in our tertiary referral hospital, but they originated from different regions in the north of the Netherlands. Spatial epidemiology data did not suggest an epidemiologic link or outbreak (data not shown). In 2 children, EV-D68 symptoms developed >48 hours after admission; the source of possible nosocomial infection was not identified.

**Figure 2:**
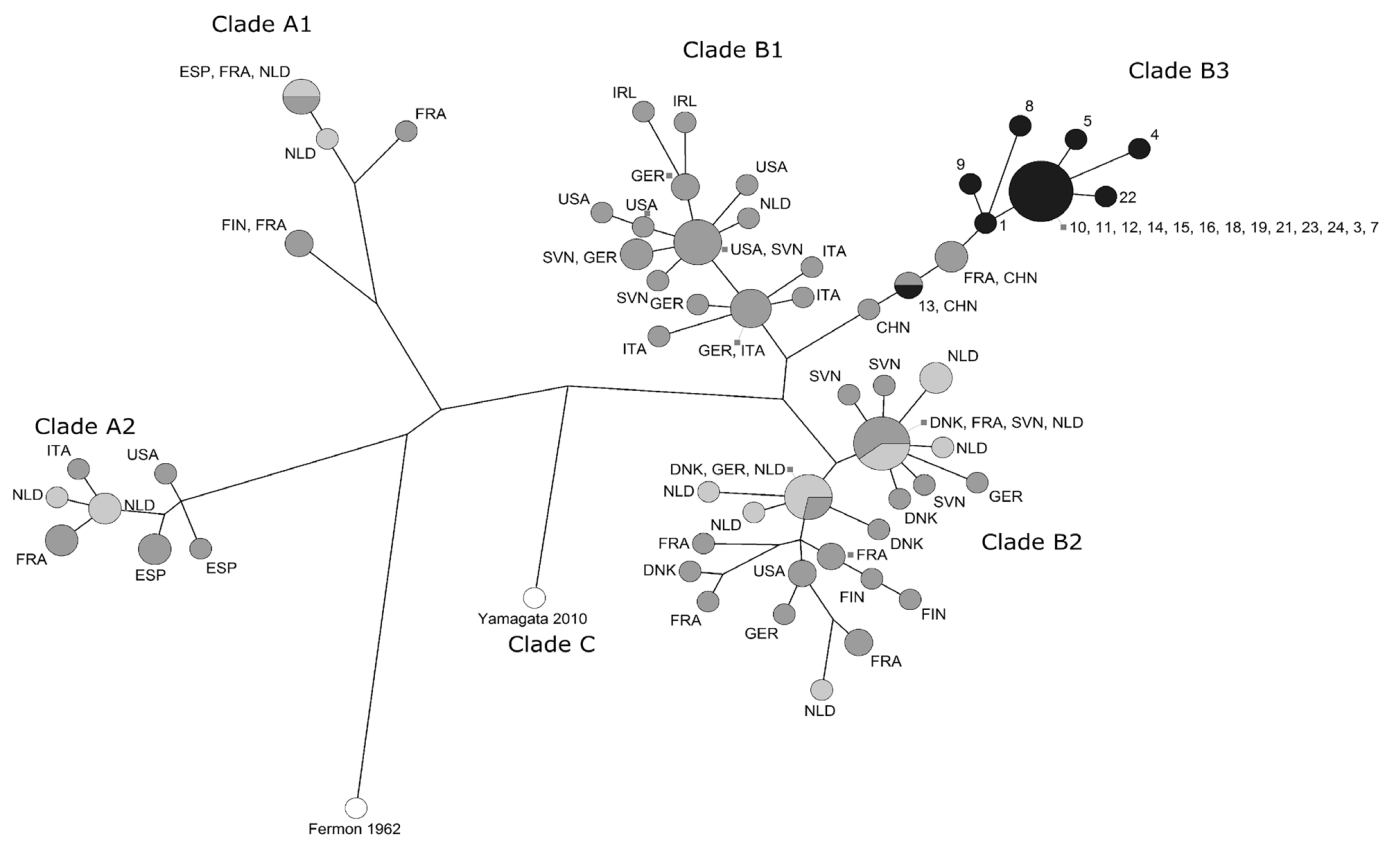
Maximum-parsimony tree of partial viral protein 1 sequences of enterovirus D68 (EV-D68). Included are the strains obtained in the laboratory of the University Medical Center Groningen (Groningen, the Netherlands) in 2014 (light gray, n = 23) and 2016 (black, n = 20) and worldwide isolates from 2014 (dark gray, n = 73). Recent strains cluster in the recently described clade B3, with a nucleotide divergence of 2.1% within clade B3, 5.5% to clade B1, and 7.3% to clade B2. Clades are according to Tokarz et al. and Gong et al (*7*,*8*). We submitted the 20 sequences we obtained during 2016 to GenBank under accession nos.: KX685066–KX685084 and KX710328. CHN, China; DNK, Denmark; ESP, Spain; FIN, Finland; FRA, France; GER, Germany; IRL, Ireland; ITA, Italy; NLD, the Netherlands; SVN, Slovenia; USA, United States. White circles indicate reference strains (n = 2).

We obtained 20 sequences of part of the EV-D68 viral protein gene and aligned them with the sequences from the 2014 epidemic. Clades were assigned as described previously (*7*) by the neighbor-joining method by using BioNumerics software 6.6 (Applied Maths/bioMérieux, Sint-Martens-Latem, Belgium). Sequence analysis (Figure 2) showed that the 2016 strains were closely related to sequences of the recently described subclade B3 (*8*), represented in Figure 2 by 4 sequences from China. The nucleotide divergence was 2.1% within B3, 5.5% between B1 and B3, and 7.3% between B2 and B3. We submitted our 20 sequences from 2016 to GenBank (accession nos. KX685066–KX685084 and KX710328).

## Conclusions

After the upsurge of EV-D68 in our region during 2009 and 2010, we set up routine genotypic characterization for several viral pathogens, including enterovirus and rhinovirus, which is also offered to regional referring hospitals and healthcare institutions. Sequencing results are available within 1 week, which provides clinically relevant and epidemiologic information. This so-called REGIOTYPE strategy contributed to timely detection of EV-D68 in our hospital in 2014, as well as in 2016. We recognized a sudden reappearance of EV-D68 with a sharp increase of cases that could not be explained by a change in surveillance strategy.

In line with previous outbreaks, mostly children were affected, and those with underlying pulmonary conditions seemed at higher risk for ICU admission (*9*). The number of children who needed ICU admission was higher in 2016 than in our 2010 and 2014 reports (*3*,*4*). Although dynamics of viral disease and shedding are not known for EV-D68, we assume a role for EV-D68 in the symptoms of patients 12 and 23, as, in line with rhinovirus infections, higher viral load might indicate symptomatic disease (*10*).

Evidence that EV-D68 might cause AFM is increasing after recent epidemiologic investigations (*1*,*11*,*12*). In patient 16, atypical Guillain-Barré syndrome initially was diagnosed; however, this diagnosis was later discarded because the electromyography results indicated motoric axon or anterior horn cell disease, and the clinical picture and MRI results were in favor of AFM (*13*). MRI findings were subtle, and radiologic diagnosis was made only after further review and discussion of the case with the neurologists. The absence of EV-D68 in CSF is consistent with previous reports (*1*,*12*).

Sequencing results showed that the strains in our study cluster in the recently described clade B3 (*8*). During the 2014 outbreak, most EV-D68 sequences belonged to clades B1 and B2, although A1 and A2 were also represented (*2*,*14*). Larger epidemiologic and genotyping studies are needed to evaluate whether the distinction within clade B is tenable and whether our clinical findings are typical for subclade B3.

This upsurge could indicate an active EV-D68 season, as highlighted by the epidemiologic curve, with a potential increase in AFM cases. Clinicians should be alert for EV-D68, its clinical implications, and the need for appropriate diagnostics, particularly in children who are admitted with respiratory failure to the ICU or with possible symptoms of AFM.
